# Genomic Characterization of a B Chromosome in Lake Malawi Cichlid Fishes

**DOI:** 10.3390/genes9120610

**Published:** 2018-12-05

**Authors:** Frances E. Clark, Matthew A. Conte, Thomas D. Kocher

**Affiliations:** Department of Biology, University of Maryland, College Park, MD 20742, USA; fclark@umd.edu (F.E.C.); mconte@umd.edu (M.A.C.)

**Keywords:** supernumerary chromosomes, B chromosomes, next-generation sequencing, coverage ratio analysis

## Abstract

B chromosomes (Bs) were discovered a century ago, and since then, most studies have focused on describing their distribution and abundance using traditional cytogenetics. Only recently have attempts been made to understand their structure and evolution at the level of DNA sequence. Many questions regarding the origin, structure, function, and evolution of B chromosomes remain unanswered. Here, we identify B chromosome sequences from several species of cichlid fish from Lake Malawi by examining the ratios of DNA sequence coverage in individuals with or without B chromosomes. We examined the efficiency of this method, and compared results using both Illumina and PacBio sequence data. The B chromosome sequences detected in 13 individuals from 7 species were compared to assess the rates of sequence replacement. B-specific sequence common to at least 12 of the 13 datasets were identified as the “Core” B chromosome. The location of B sequence homologs throughout the genome provides further support for theories of B chromosome evolution. Finally, we identified genes and gene fragments located on the B chromosome, some of which may regulate the segregation and maintenance of the B chromosome.

## 1. Introduction

The genomes of eukaryotic species are typically organized into linear chromosomes, and each species has a characteristic number of chromosome pairs referred to as the A chromosomes (As). The genomes of at least 2828 eukaryotic species contain additional chromosomes commonly referred to as B chromosomes (Bs) [1]. These supernumerary B chromosomes are not essential and are found in some but not all individuals of a population [2,3,4,5]. Among species, the number of B chromosomes in each cell has been found to vary from 1 to 50 [6,7,8]. B chromosomes are thought to manipulate the normal mechanisms of cell division in order to increase their transmission to the next generation, a process known as drive [4,7,9].

B chromosomes often contain large amounts of highly repetitive DNA [5,10,11,12] and are frequently either partially or completely heterochromatic [3,5,7]. In several species, it has been shown that B chromosomes share homology with sequences from all or many of the A chromosomes [13] (the grasshopper *Podisma kanoi* [11], the fish *Astatotilapia latifasciata* [14], rye *Secale cereale* [15], and maize *Zea mays* [10]). This suggests that sequences on B chromosomes are derived from the A chromosomes through as yet uncharacterized mechanisms of gene duplication [16]. Theoretically, because they are nonessential, B chromosomes should experience relaxed selective pressures [16,17]. For this reason, they might be expected to experience high rates of sequence turnover. B chromosomes are continuously acquiring new sequences. Sequences already on the B collect mutations at a high rate, and most are eventually lost. It has been difficult to produce sequence assemblies of B chromosomes due to their repetitive nature and their high levels of homology with sequences in the A chromosomes [18,19,20,21].

Despite the fact that B chromosomes add significant amounts of genetic material to the genome, B chromosomes have rarely been associated with novel phenotypes, the most frequent exception being an effect on fertility [3,7,22,23,24]. With a limited list of known B-specific sequences and few or no visible phenotypes beyond drive, the prevalent view has been that B chromosomes carry few genes [16,25]. They have been thought to be composed of nonfunctional “junk” DNA together with one or two genes contributing to drive [11]. 

Recent advances in next-generation sequencing and bioinformatic analyses of genomic data have begun to contradict this long-standing view. These technological and analytical improvements make it possible to address many questions about B chromosome biology, including how Bs acquire sequence from the As, how these sequences evolve once on the B chromosome, whether and to what extent the B contains functional sequence, and finally, the identification of the gene(s) controlling drive. Examples of genic sequences detected on B chromosomes include the C-KIT gene in two *Canidae* species [26], ribosomal RNA (rRNA) genes and thousands of genes and gene fragments in the fish *Astatotilapia latifasciata* [14,27], protein coding genes in the grasshopper *Eyprepocnemis plorans* [28], and protein-coding genes in two mouse species from the genus *Apodemus* [29]. Furthermore, transcription has been characterized for an rRNA gene in the smooth hawksbeard *Crepis capillaris* [30], rRNA genes and a pseudogene in the grasshopper *E. plorans* [31,32,33], pseudogenes in rye *Secale cereale* [34], and protein coding genes in maize *Zea mays* [35,36]. 

Current approaches to identifying B sequences can be categorized into two types: direct and indirect [18]. Direct methods, such as the sequencing of B chromosomes isolated through flow sorting or microdissection, have a high rate of contamination [14,18] and are only possible in a few organisms. Indirect methods, such as the comparison of whole genome sequence data between samples with or without a B chromosome, can be performed on any species. For many species, the sequence reads can be aligned to a reference genome assembled from an individual lacking a B chromosome, allowing a characterization of a B sequence by its alignment to homologous portions of the A genome. While Illumina sequencing has dramatically lowered costs, there are considerable limitations to Illumina sequence data [37]. Namely, Illumina reads are very short and are not very useful for assembling the repetitive sequence of B chromosomes. However, the extent to which short reads can be used to identify B chromosome sequence has not been fully explored.

Among cichlid fishes, B chromosomes were first identified in species from South America [38,39,40]. More recently, they have been identified also in species from lakes Victoria and Malawi in East Africa [41,42]. B chromosomes have been found in at least seven species from Lake Malawi. In all seven species, B chromosomes are found only in females, but not all females have a B chromosome. The females that do possess a B chromosome have only a single B (haploid) per cell [42]. A karyotype of one of these species, *Metriaclima lombardoi*, shows the B chromosome is one of the largest chromosomes, representing approximately 4.5% of the genome when present. In Lake Victoria cichlids, B chromosomes are found in both males and females, and individuals carry as many as three B chromosomes per cell [41,43]. Whole-genome resequencing and the sequencing of a microdissected B chromosome were used to identify B chromosome sequences in the Lake Victoria species *A. latifaciata* [14]. Mapping of whole genome sequencing reads to a reference assembly identified thousands of gene fragments and tens of complete genes on the B chromosome. Sequence of microdissected B chromosomes detected only a small portion of the overall B chromosome in this study. Presumably, the sequences contributing to drive are among the genes and gene fragments identified in this study. 

Here, we performed a sequence coverage ratio analysis of multiple individuals and species from Lake Malawi to systematically detect sequences from the B chromosome. From this, we identified genes and gene fragments on the B chromosome. We characterized the location and length of the homologous A-located sequences to understand the origin and dynamics of sequence accumulation on the B chromosome. Finally, we analyzed the proportion of B chromosome sequences that are shared among species to estimate the rate of sequence turnover on these unique chromosomes.

## 2. Materials and Methods

All procedures involving live animals were approved by the University of Maryland IACUC and conducted in accordance with protocol #R-13-58. The *M. lombardoi* individuals used were collected from stocks maintained at the Tropical Aquaculture Facility at the University of Maryland. These stocks were originally sourced from Lake Malawi, Africa in 2014–2016. The remaining individuals were collected directly from Lake Malawi in 2005, 2008, and 2012. Individuals were euthanized using tricaine methanesulfonate (MS-222) and inspected for testes or ovaries to confirm sex. Standard phenol chloroform methods were used in conjunction with phase-lock gel tubes (5Prime, Gaithersburg, MD, USA) for DNA extraction from fin tissue. Genotyping of B-specific single nucleotide polymorphisms (SNPs) was performed according to [42] to identify individuals carrying a B chromosome. Blood was collected from a *M. lombardoi* individual in order to prepare the high molecular weight DNA necessary for Pacific Biosciences SMRT (PacBio, Menlo Park, CA, USA) sequencing.

Illumina sequencing was performed from the fin clips of 12 female individuals with a B chromosome. To provide a comparison lacking B chromosome DNA, the sequence from pooled male individuals, previously collected and sequenced with Illumina (San Diego, CA, USA), was used. Each pooled sample contained between 10 and 20 male individuals of that species. As there was no male *M. lombardoi* sequence data available, the B female *M. lombardoi* data was compared to a pool of *Metriaclima zebra* “Boadzulu” males for the scaled coverage analysis. Sequences from two samples of pooled female individuals lacking a B chromosome (NoB), previously collected and sequenced with Illumina, were used as controls. These two NoB samples represent two types of females from the *Labeotropheus trewavasae* “Maison Reef” population, XX females, and WZ females, all lacking a B chromosome. The samples used are summarized in Table 1.

The 12 B female individual samples, the 7 NoB male pooled samples, and the 2 NoB female pooled samples were prepared for Illumina sequencing with the TruSeq DNA sample preparation kit ver.2 rev.C (Illumina Inc.). Each DNA sample was sonically sheared and selected to produce libraries of 500 bp fragments. Paired-end reads of 100 bp were obtained using an Illumina HiSeq 1500. Pacific Biosciences SMRT sequencing was performed on one *M. lombardoi* B female. DNA was extracted from nucleated blood cells using the MagAttract HMW DNA kit from Qiagen (Germantown, MD, USA). Pulse-field gel electrophoresis was performed with a Blue Pippin instrument by the University of Maryland Genomics Resource Center to select DNA fragments of the proper size. PacBio sequencing was carried out on the PacBio RS II platform with P6-C4 chemistry using nine SMRT cells and on the PacBio Sequel platform using nine additional SMRT cells. Illumina and PacBio sequencing reads were aligned to the reference assembly of a *M. zebra* “Mazinzi Reef” NoB male individual sequenced with PacBio [44], (publicly available on NCBI, Accession: GCA_000238955.4, [45]) with BWA [46] and NGM-LR [47], respectively. BWA alignments were then run through Picard (version 2.1.0) “MarkDuplicates” (http://broadinstitute.github.io/picard) to identify PCR duplicates.

After alignment to the reference genome, all genomic samples were analyzed with samtools (version 0.1.18) mpileup (http://samtools.sourceforge.net) to calculate read coverage depth across the genome. The raw coverage depth was scaled by dividing the raw coverage at each position by the average genome-wide coverage depth of the sample. This scaled coverage value was then used to calculate the scaled coverage ratio (SCR) between the B chromosome female and the corresponding NoB pooled male sample:

(1) $$\mathrm{SCR}=\frac{\mathrm{scaled}\;\mathrm{coverage}\;\mathrm{of}\;\mathrm{the}\;B\;\mathrm{female}}{\mathrm{scaled}\;\mathrm{coverage}\;\mathrm{of}\;\mathrm{the}\;\mathrm{NoB}\;\mathrm{male}\;\mathrm{pool}}.$$

For each base in the genome, a binomial test was performed to check for a statistically significant difference in coverage between the B female dataset and the NoB pooled male dataset:

(2) $$P\left(X\right)=\frac{n!}{\left(n-X\right)!X!}\cdot {\left(p\right)}^{X}\cdot {\left(q\right)}^{n-X.}$$

In this binomial test, *X* represents the raw coverage depth in the B female sample and *n* is the sum of the raw coverage depth in the B female sample and the NoB pooled male sample. The expected frequency of B female reads, *p*, is calculated from the relative genome-wide sequence depth of the B female sample. The expected frequency of NoB pooled male reads, *q*, is calculated from the relative genome-wide sequence depth of the NoB pooled male sample. Any positions with a SCR ≥ 3 (corresponding to ≥4 B-located copies), a binomial test *p*-value ≤ 0.001, and within 300 bp of another such position were merged into a block feature with Bedtools (version 2.26.0) merge function [48]. Requiring a minimum SCR of three fails to detect any sequences with fewer than four copies on the B chromosome avoids detection of simple A chromosome duplications, which would result in a SCR of 2. These block features were filtered to remove any block feature ≤ 500 bp in length and then any block feature with ≤10% of the positions spanned meeting the SCR ≥ 3 requirement and the *p*-value ≤ 0.001 requirement. The latter three parameters (merging distance of 300 bp, minimum block length of 500 bp, and minimum percent positions of 10%) were chosen after manual inspection of several preliminarily identified regions. The remaining block features are referred to as “B blocks”. The B blocks of all individuals were then processed with Bedtools (version 2.26.0) intersect [48] to find B blocks common among at least 12 of the 13 B individuals (12 Illumina and 1 PacBio). These shared B blocks are referred to as the Malawi “core” blocks.

The sum of the lengths of all B blocks was calculated as an estimate of total B sequence length in the reference genome, further referred to as “A chromosome space”. To account for copy number of these sequences on the B, the length of each block was multiplied by its estimated copy number, resulting in each block’s contribution to the B, which was then summed to estimate the total B sequence length. Estimated copy number was calculated with one of two equations, depending on the average scaled coverage in the NoB male dataset. For NoB male scaled coverage ≥1, we used Equation (3):

(3) (SCR ∗ 2)−2

In Equation (3), SCR was multiplied by 2 to compensate for the fact that we are comparing a haploid B genome to a diploid A genome. The A chromosome copy was then accounted for by subtracting 2. To avoid overestimating the B-located copy number when the NoB male scaled coverage was less than 1, we used Equation (4):

(4) Female Scaled Coverage ∗ 2

Here, a NoB male scaled coverage of 1 was assumed (accounting for one copy of this sequence in the A genome of the reference), allowing us to use the scaled coverage of the B female to estimate copy number, without having to account for the A chromosome copy by subtracting 2. Example scripts for our B block identification analysis are provided in Supplementary Materials (Directory S1).

## 3. Results

### 3.1. Identification of B Chromosome Sequence

#### 3.1.1. Characterization of B Blocks

**Figure 1:**
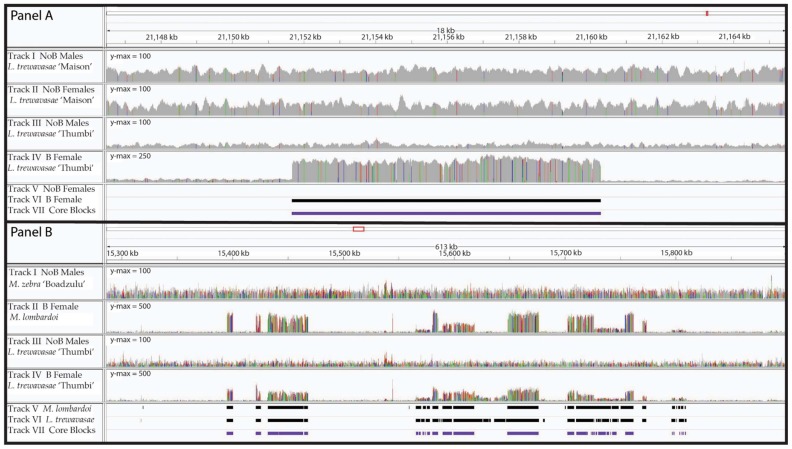
Read Coverage and B Blocks. B blocks from two genomic regions are shown with the corresponding read coverage. **Panel** (**A**) depicts an 18-kb region of LG8 with a typical B block. Tracks I and III are the male coverage (*Labeotropheus trewavasae* “Maison” and *L. trewavasae* “Thumbi”, respectively), while tracks II and IV are the female coverage (NoB XX *L. trewavasae* “Maison” control and B *L. trewavasae* “Thumbi”, respectively). Please note the *y*-axis maximum is 100 for tracks I, II, and III but 250 for track IV. Beneath the coverage plots are the blocks detected by our analysis; track V shows the NoB XX female blocks, track VI shows the B female *L. trewavasae* blocks, and track VII shows the core blocks. A ~8.5-kb B block can be observed by the increased coverage in the B female *L. trewavasae* (track IV), but no such increased coverage is observable in the other coverage plots. Our B block analysis pipeline identified the B female *L. trewavasae* block (track VI) but did not identify a block in the NoB XX female control data (track V). As this B block was similarly found in at least 12 of the 13 datasets, it is included in the core block set (track VII). **Panel** (**B**) depicts several B blocks in close proximity to one another across a 613-kb region of LG23. Tracks I and III are again male coverage but for *M. zebra* “Boadzulu” and *L. trewavasae* “Thumbi”, respectively. Tracks II and IV both depict B female coverage (B *Metriaclima lombardoi* and B *L. trewavasae* “Thumbi”, respectively). Please note the *y*-axis maximum is 100 for tracks I and III but 500 for tracks II and IV. The block sets detected in the B female *M. lombardoi* (track V), B female *L. trewavasae* “Maison” (track VI), and the core blocks (track VII) are shown below. B blocks can be observed in the coverage of both B females (tracks II and IV) and correspond well with the blocks identified through our B block identification analysis (tracks V, VI, and VII). The B blocks span ~420 kb and appear to have migrated to the B as a single unit in the ancestor of *M. lombardoi* and *L. trewavasae*.

Due to the homology between A and B chromosome sequence, most sequence reads derived from the B chromosome will align to their A chromosome homologs present in the reference genome. As a result, alignments of reads from a genome with a B chromosome will have regions of increased coverage compared to an alignment from a genome lacking a B. Our analysis of coverage ratios initially identified 0.34%–1.31% of the bases in the genome as having relatively higher coverage in the B female dataset (Table 2). In comparison, the same analysis in our controls identified 0.06% and 0.44% of bases in the WZ and XX NoB females, respectively. Further analysis combined these individual bases into features referred to as B blocks, defined as consecutive sequence with increased coverage in B chromosome samples. Thousands of B blocks were identified in each B female individual. B blocks ranged in length from 500 to 100 kb, although there were multiple regions in the genome with multiple B blocks in close proximity, suggesting that a larger region was transferred to the B chromosome as a whole (Figure 1). The largest such regions were located on LG4 (~120 kb), LG9 (~250 kb), LG17 (~260 kb), and LG23 (~420 kb). 

**Figure 2:**
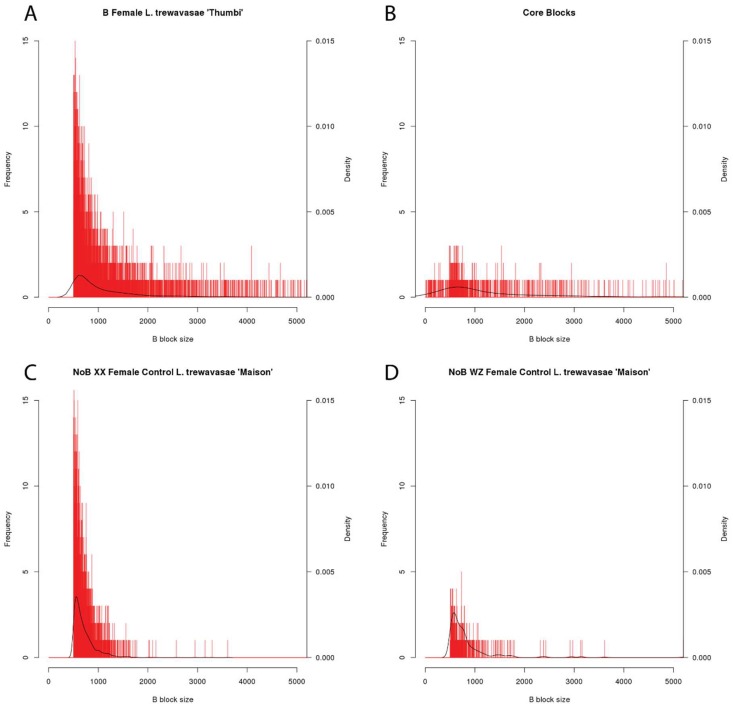
Block Length Histograms. Histograms of B block length for four datasets. B block size along the *x*-axis is reported in bp (bin size = 1 bp), and the *x*-axis maximum is 5000 bp to more easily view the majority of the data. The blocks not visible in this graph, larger than 5000 bp, represent only 10.3% of the core blocks and <5% of the blocks in the B female and two NoB controls. The number of blocks of each length is shown with red bars with the *y*-axis scale on the left and the density is depicted with a black line with the corresponding *y*-axis scale on the right. Because blocks shorter than 500 bp were removed during analysis, the B female (**A**) and the two NoB controls (**C**,**D**) show a lack of these smaller blocks. However, during the identification of core blocks, some larger B blocks were fragmented further, resulting in the smaller B blocks shown in the core block set histogram (**B**). All other B block length histograms are included in the Supplementary Materials as Figure S1.

In the WZ and XX NoB females controls, we identified 343 and 2125 putative B blocks, respectively, and the longest blocks were only 3.6–5.2 kb (Table 2). As neither of these individuals carried a B chromosome, these putative B blocks represent false positives. While actual variation in A genome copy number may explain some of this error, stochastic variation in the coverage depth of Illumina data and regions of poor alignment likely also contribute to these false B block calls. Figure 2 provides representative histograms of block length, showing data for a B chromosome female (*L. trewavasae* 2005-1306), the blocks included in the core set, and the XX and WZ NoB females. Both the B female and the core set show enrichment for blocks of longer lengths when compared to the controls. The core set shows a depletion of shorter blocks. An interpretation of this is that false positive B block calls are more likely to be short in length and that a sizable portion of the shorter B blocks may be false positives (type 1 error) and do not represent actual B sequence. However, since large regions, as seen in Figure 1, are often fragmented into smaller block calls, we opted not to remove the shorter block calls at this stage of the analysis. The B block information for each dataset, including block location, coverage details, and length, is provided in Supplementary Materials (Directory S2).

The lengths of all B blocks were then summed for each sample, as well as for the set of core blocks, producing the total length of B sequence in A chromosome space (Table 3). However, since there are multiple copies of these sequences on the B, we multiplied the length of each block by the copy number of that sequence, as estimated by the difference in coverage between the B female dataset and the male dataset. These values were then summed across all blocks to produce the total estimated length of B chromosome sequence (i.e., in B chromosome space). The total length of B sequence from the core block set (not including variable blocks specific to some individuals or species) in B chromosome space was also calculated for each sample.

The total length in A space ranges from 3.51 to 14.06 Mb among the B females and only 0.28 to 1.52 Mb in the controls. Only 1.37 Mb (in A space) is shared among at least 12 of the 13 B females. After taking copy number of these sequences into account, the total length in B space ranges from 23.19 to 99.69 Mb among B females and only 0.39 to 2.15 Mb in the controls. The 1.37 Mb of core blocks in A space translates to 12.31–44.07 Mb among B females and as little as 0.63–0.80 Mb in the controls. 

The consensus, or core, block set with blocks common to at least 12 of the 13 individuals successfully removed the greatest proportion of false positives (type 1 error). However, the core block set lacks any B chromosome sequence that is specific to only a few individuals or species. The B chromosome of the *M. lombardoi* individuals, sequenced with Illumina, is estimated to be 58.48–74.53 Mb in length. Considering just the most conservative B blocks (the core set), the estimated length is 17.67–24.09 Mb in these individuals. Karyotype data, available only for *M. lombardoi*, shows that the B chromosome is one of the largest chromosomes. A tentative estimate of chromosome size from karyotype data suggests a B chromosome of roughly 50 Mb. The total length of B sequence in B space in these three individuals may be inflated by false positive blocks, while the total length of core sequence is B space is slightly smaller than the length estimated from the karyotypes. The variation in estimated B chromosome length across individuals could indicate that B chromosomes vary in size among these species. This is consistent with the finding that B chromosomes vary in length within and among species of Lake Victoria cichlid. [43]. Notably, *Melanochromis auratus* consistently has the least amount of sequence detected by our analysis. The 12.31 Mb, in B space, found in *M. auratus*, compared to the 30.84 Mb found in *Metriaclima greshakei*, suggests that the B chromosome of *M. greshakei* may be twice as large as the B chromosome of *M. auratus*.

#### 3.1.2. Comparison of Illumina and PacBio Sequence Data

To better understand the differences in B blocks called from Illumina and PacBio datasets, we compared an *M. lombardoi* B female sequenced with PacBio to the three *M. lombardoi* B females sequenced with Illumina. The Illumina reads are 100 bp in length and the PacBio reads averaged 8295 bp. The blocks identified in the individuals sequenced with Illumina ranged in total length in A space from 10.73 to 14.06 Mb, whereas the total length of blocks identified in the individual sequenced with PacBio was only 5.66 Mb in A space. As demonstrated with the block size histograms (Figure 2), we believe most falsely identified blocks are short in length. Indeed, the mean length of B blocks identified using the PacBio data was much longer than with the Illumina data (Table 2) and a depletion of shorter blocks can also be seen in the block size histogram of the PacBio data (Supplementary Materials, Figure S1). This discrepancy in length in A space could be a byproduct of the longer PacBio reads resulting in more consistent coverage and preventing the erroneous identification of shorter blocks. Additionally, longer PacBio reads will have more accurate mapping in repetitive regions than the shorter Illumina reads. These factors suggest that PacBio data would result in fewer false positives or type 1 errors. However, even when using the conservative core block set, the PacBio data identified only 15.02 Mb of core sequence in B space compared to the 17.67–24.09 Mb identified in the three Illumina datasets, suggesting the Illumina data is able to detect sequences the PacBio data does not.

**Figure 3:**
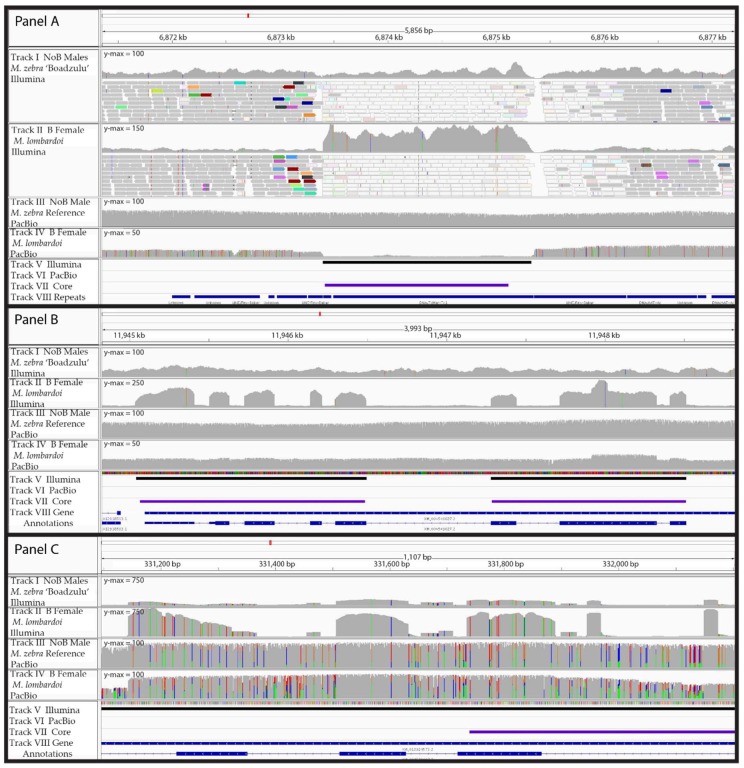
Comparisons of Illumina and PacBio read alignment in B blocks. Differences in the alignment of Illumina and PacBio reads affected the B block identification analysis. Panels (**A**–**C**) represent regions of LG20, LG22, and the unanchored scaffold 000256F_pilon_quiver, respectively. Panel (**A**) demonstrates a failure to identify a B block with PacBio data. Additionally, the localization of that block with Illumina data to a recent insertion inaccurately suggests LG20 as the A chromosome origin of this B-located sequence. Panel (**B**) demonstrates the failure of PacBio data to detect a retrogene. Panel (**C**) demonstrates a case where Illumina data suggests a retrogene, which the PacBio data reveals to be a complete gene (possessing both exons and introns). This specific example also shows increased coverage in the NoB data, suggesting the region has also experienced a duplication event within the A genome in addition to the copies present on the B chromosome. In Panels (**A**–**C**), tracks I and II represent the coverage of the NoB male and B female sequenced with Illumina, respectively, while tracks III and IV depict the coverage of the NoB male and B female sequenced with PacBio, respectively. In Panels (**A**–**C**), the B female *M. lombardoi* block sets for Illumina and PacBio are shown in tracks V and VI, respectively, while the core block set is shown in track VII. Tracks I and II in Panel (**A**) also show a portion of the reads aligning to that region. Reads shown in white have a map quality of 0 indicating multiple mapping to several regions. In Panel (**A**), track VIII displays the annotated repeat content of this region. In Panels (**B**,**C**), track VIII displays the gene annotations. Please note the *y*-axis maximum of the coverage plots varies to best view the variable coverage data of each plot.

While inspecting the read alignments and coverage data in detail, a few key patterns emerged. First, there were several regions of high coverage in the Illumina data, which had low coverage in the PacBio data (Figure 3, panel A). The Illumina reads in these short regions all aligned to several other locations (as indicated with white reads in Figure 3) and these regions were annotated as various repeats. Our interpretation is that these regions represent a shorter, highly repetitive sequence, with many copies found on the B chromosome. We hypothesize that the A chromosome in the *M. zebra* reference assembly experienced a recent insertion of this repeat, resulting in a lack of coverage by the *M. lombardoi* PacBio data because it does not have this insertion. Because the Illumina reads are too short to span the length of the repeat, they aligned to this insertion in the reference. This means that the Illumina data was able to detect these B-specific sequences while the PacBio data was not. However, the Illumina data wrongly places the A chromosome origin of these B sequences at the new insertion site when their existence on the B appears to predate this insertion.

A second difference between the sequence data types was in the detection of retrogene insertions (Figure 3, panel B). Again, since the PacBio reads are much longer than the retroinserted exons, they do not align well to the A reference using typical PacBio alignment software such as NGM-LR and BLASR with standard alignment parameters. In contrast, Illumina reads are usually shorter than the length of these retroinserted exons and therefore do align well to the reference. This means that standard alignment software and parameters will detect retroinserted sequences on the B chromosome with short read data but not with long read data. Proper alignment of retroinserted genes using PacBio reads requires the use of alignment tools that are splice-site aware, such as GMAP. We were able to recover this particular retroinsertion with the PacBio data by aligning with GMAP, but the majority of A genome reads did not map. Alignment software that accounted for both types of reads is needed, but to our knowledge, such tools do not yet exist. 

The third difference between the two sequence data types was in the false detection (type 1 error) of retroinserted genes (Figure 3 panel C). The Illumina data showed increased coverage in the exons but not the introns of some genes, suggesting it was another retroinserted gene on the B. However, the PacBio data revealed consistently high coverage across both introns and exons, with much higher sequence polymorphism in the introns. The higher sequence polymorphism in the introns compared to the exons suggests that the B-located copy of this gene is relatively old and still experiencing purifying selection for the encoded protein. The short reads of the Illumina data failed to align to the divergent introns but did align in the less divergent exons, resulting in what appeared to be a retroinserted gene. We were only able to distinguish between ‘true’ and ‘false’ retroinserted genes on the B chromosome by comparing the Illumina data with PacBio data.

### 3.2. B Block Turnover

B chromosomes are thought to have a high rate of sequence turnover because they experience little purifying selection [16,17]. Because the Lake Malawi cichlid species studied here diverged less than 1 million years (MY) ago [49], we have an opportunity to study the rates and patterns of sequence turnover on the B chromosome. To gauge the amount of sequence turnover that has occurred between these species, we compared the core block set to all B blocks (core and variable) identified in each individual. The core blocks accounted for 30.22%–53.63% of total B sequence (in B space), leaving 46.37%–69.78% B sequence (in B space) variable among individuals. While some of the variable B blocks represent false positives (type 1 error), many represent sequences that are unique to a particular individual or species. These variable B blocks likely represent both sequence that was lost from a common ancestor and a new sequence acquired during the evolution of particular lineages.

### 3.3. B Block Origin

**Figure 4:**
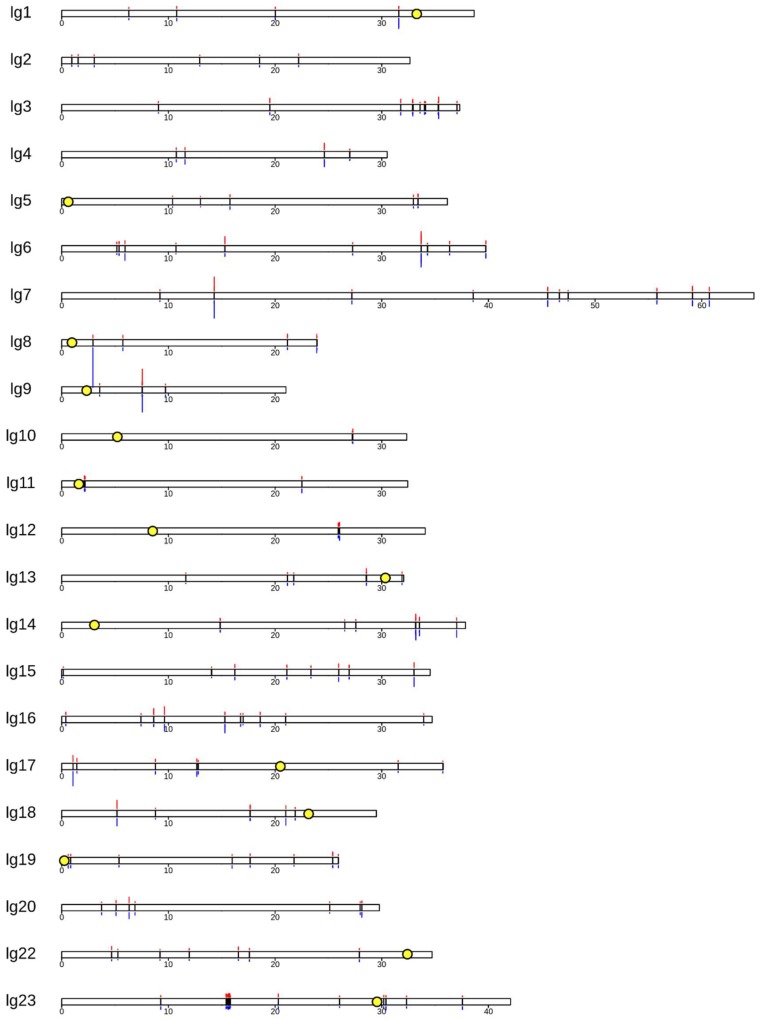
Karyoplot showing the A genome origins of the B chromosome. The position of B blocks (black bars) is superimposed on a karyoplot of the *Metriaclima zebra* “Mazinzi” reference genome. The A genome consists of 22 chromosomes. For simplicity, unanchored scaffolds of the genome assembly were not included. Physical distances are noted beneath each LG in Mb and the locations of centromeres (available for only some LGs) are indicated with yellow circles. Above and below each LG is a bar graph representing the scaled coverage ratio (SCR) of each core block. Above, in red, is the SCR of *M*. *lombardoi* 2014-1021. Below, in blue, is the SCR of *L*. *trewavasae* 2005-1306. The SCR of these individuals ranges from 3 to 234. The two individuals shown are arbitrarily chosen representatives of these two genera. This plot was created with the R package KaryoploteR [50].

A comparison across these 13 B individuals has allowed us to identify sequence (the core blocks) present on the B chromosome of the most recent common ancestor to these seven cichlid species. Figure 4 depicts the position of core B blocks on the chromosome-scale assembly of the *Metriaclima zebra* A genome. Notably, each linkage group (LG), and therefore each chromosome, has at least one core B block and most have several, distantly spaced core B blocks. This is consistent with the idea that cichlid B chromosomes continue to collect A chromosome sequences over time [13,14,15]. No trend was observed between B block position and centromere position. There is no readily visible pattern, suggesting certain regions are more likely than others to be the source of B chromosome sequence. The longest stretch of B chromosome (along A chromosome space) corresponds to a ~420-kb region comprising several neighboring B blocks on LG23 (also shown in Figure 1). The SCR of the core blocks varies among individuals. The largest difference in SCR between these two individuals is shown on LG8.

### 3.4. Genes

B chromosome gene sequences were identified as overlap between RefSeq annotated genes and B blocks. Annotated genes were either partially or completely encompassed in a B block. The total number of partial or complete genes in the B chromosome blocks is listed in Table 4. The complete list of genes and gene fragments identified in each dataset is provided in Supplementary Materials (Directory S3). 

**Figure 5:**
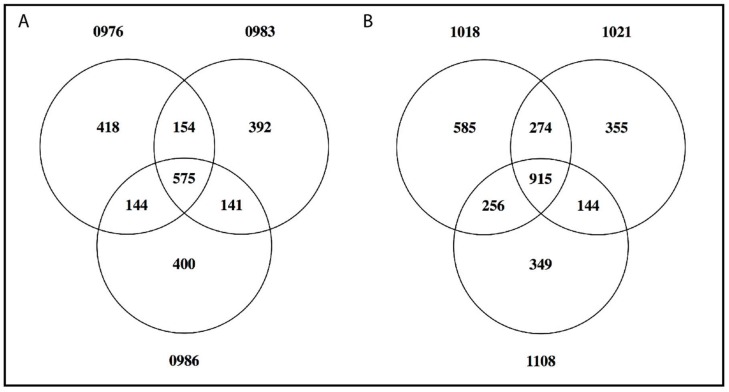
Shared B-located genes and gene fragments among individuals of the same population. The Venn diagrams show the number of genes and gene fragments shared by (**A**) three *M. zebra* “Nkhata Bay” individuals and (**B**) three *M. lombardoi* individuals.

Figure 5 includes two Venn diagrams of B-located genes shared among the three *M. zebra* “Boadzulu” individuals (0976, 0983, and 0986) and among the three *M. lombardoi* individuals (1018, 1021, and 1108). In both cases, the individuals from the same population share most of their presumed B-located genes, though there are still several hundred unique to each individual.

## 4. Discussion

Using an analysis of sequence coverage, we identified 1.37 Mb of the A genome that has been copied to the B chromosome and which is now shared among several Lake Malawi cichlid species as core B chromosome sequence. In addition to this core sequence, there were many additional Mb of B chromosome sequence that were found among various subsets of individuals/species. Because the core B chromosome sequences are found in multiple copies, the total length of B-specific sequence in the three *M. lombardoi* individuals totaled 17.67–24.09 Mb. This is consistent with the size of the *M. lombardoi* B chromosome observed in karyotype data. This suggests that the coverage ratio analysis was successful in identifying an appreciable amount of sequences on the B chromosome. Using all the B blocks identified with each individual dataset (including both variable and core blocks) resulted in a size estimate of 58.48–74.53 Mb (in B space), which is slightly larger than expected from karyotype data. This suggests that some portion of the identified B blocks represent false positives, or type 1 error. Another approach to understanding the amount of type 1 error in this analysis is through the two control datasets. The percentages of individual bases in the genome passing the SCR and binomial thresholds were not markedly different for the XX NoB female control (0.44%) and the B females (0.34%–1.31%). Our downstream filtering to produce block features helped to further reduce the type 1 error, resulting in an order of magnitude fewer blocks identified in the two controls compared to the B female datasets. Further filtering of short blocks would likely continue to reduce the type 1 error but would simultaneously increase type 2 error. The length of identified B sequence, in B space, of the two controls was 0.39–2.15 Mb. Arguably, we can extrapolate from this to predict that any individual could have at least 1–5 Mb of falsely identified B sequence. Yet, the total amount of variable B sequence, in B space, ranged from 39.58–50.45 Mb for the B female datasets. From this, we conclude that B blocks identified using sequence data from a single individual likely contain some type 1 error but also correctly represent a large number of unique B blocks that are not shared among individuals or species.

In the estimation of total length in B space, proper estimation of B-located copy number is clearly crucial. For most regions, SCR can be used to estimate copy number. However, in regions of poor alignment, scaled coverage can be <1, leading to an overestimate of copy number and therefore an inflated estimate of total length in B space. To avoid this issue, the B-located copy number of any region with a scaled coverage value <1 in the NoB male dataset was instead calculated with the scaled coverage in the female B dataset. The use of multiple individuals and the identification of core sequence greatly reduces the type 1 error and we suggest that multiple individuals, if not species, be used to produce the most conservative identification of B sequence when using a coverage ratio analysis. Notably, our coverage ratio analysis ignores any sequence entirely unique to the B chromosome (not aligned to homologous A sequence) or any sequence with fewer than four B-located copies (an SCR of 3). While unique sequences are mechanistically entirely undetectable with a coverage ratio analysis, the detection of less abundant sequences on B chromosomes presents a trade off with type 1 error rates. This can be circumvented by sequencing individuals with a high number of B chromosomes per cell, when possible.

Across the 13 datasets, the core blocks represented 30.22%–53.63% of the total sequence identified in B space, leaving 46.37%–69.78% as variable among individuals. As discussed above, we believe an appreciable amount of this unshared sequence is actually type 1 error, and therefore more than 30%–54% of the B is shared among these species. Even though the individuals in this study span three genera, they are less than 1 MY diverged from one another. This suggests that the Lake Malawi B chromosome has experienced turnover of roughly half of its sequences in 1 MY. Whether the rate of sequence turnover is constant or varies over time is not yet known.

The length and position of B blocks along the A chromosomes allows us to begin unraveling the history of the B chromosome. The presence of B blocks on every chromosome supports the idea that once a proto-B forms, it somehow acquires sequence from the rest of the genome. How these sequences make their way to the B, and which types of sequences are most likely to do so, is still unknown. Most discussion of mechanisms that transfer sequence to the B involves transposable elements [9]. It is possible other mechanisms, such as nonhomologous recombination, could also be contributing to the acquisition of B sequence. B blocks range in size from a few hundred to a few hundred thousand bases. Homologous regions larger than 100 kb have been found on each of several chromosomes, suggesting that some, if not all, of these larger regions must have migrated to the B after its origin. So, the mechanisms responsible for the migration of sequences to the B must include a mechanism capable of moving and incorporating sequence blocks greater than 100 kb. While not common, transposable elements are known to move such large regions [51]. These large regions are not restricted to the distal chromosome arms, as would be expected if translocations were responsible. Furthermore, the core blocks appear to be evenly distributed across the LGs, suggesting that location along the A chromosome does not impact likelihood of migration to the B. Of course, if multiple mechanisms are involved in the acquisition of A sequence, the combination of blocks acquired via these multiple methods might obscure actual patterns in the block location data.

The most extreme divergence in SCR of core blocks between the two individuals is found on LG8 (Figure 4). The SCR of this core block is 17.5 in the *M. lombardoi* B female and 234 in the *L. trewavasae* B female. This illustrates that copy number can vary greatly and is not an indication of how long a sequence has been on the B chromosome. We suggest caution in making interpretations about the origins of the B chromosome from observations of the length and position of B blocks or their copy number on the B chromosome. In these cichlids, the longest regions of homology are dispersed over too many chromosomes to suggest they were all involved in the production of the proto-B. Similarly, regions with some of the highest SCR, therefore contributing a significant amount of sequence to the B chromosome, are regions with relatively low SCR in other species. Moreover, the rate of Malawi cichlid B sequence replacement suggests that any B chromosome more than a few million years old may have replaced the original sequence of the proto-B to the point that none remain, making assignment of origin impossible. We suggest that efforts to identify the origin of B chromosomes focus on very young B chromosomes, and then use a combination of basic sequence homology, as performed here, as well as approaches that study chromosomal rearrangements and/or centromere evolution. 

The number of genes and gene fragments overlapping with B blocks ranged from 516 to 2030 among datasets. Only 132 were common to at least 12 of the 13 datasets. When comparing individuals of the same population (Figure 5), the majority of genes identified were shared. However, several hundred genes were still unique to one or two of the individuals. We believe this is the result of the higher amount of type 1 error in the unique, unshared B blocks. Again, the core blocks provide us with the most conservative estimate of gene number. Furthermore, the comparison between Illumina and PacBio datasets revealed that some blocks, while representing B sequence, are erroneously positioned in the A reference where a recent insertion of that repeat occurred. If such an insertion were to occur in the intron of a gene, our analysis would incorrectly identify that gene as being partially on the B chromosome, leading to an overestimate of gene number. Nevertheless, if the B chromosomes of these different species use the same gene(s) to achieve drive, it is reasonable to believe that gene might be found among the 132 genes common across species.

Our analysis has identified both genes and gene fragments indiscriminately. The question remains whether these genes are functional or merely pseudogenes. While it may be tempting to label the gene fragments as pseudogenes, we do not know the structure of these sequences on the B chromosome. These gene fragments may be part of a gene fusion on the B, active but with an altered function. Moreover, transcription of altered (truncated, or partially deleted) copies of these genes could function by interfering with the activity of the original gene. Further examination of the genes on B chromosomes is needed before any conclusion regarding the functionality, or lack thereof, of B-located genes. A study of B sequence function will also serve to indicate which genes among these 132 could control B chromosome behavior, namely, drive and female sex bias. A more complete understanding of the structure of the B, rather than a series of fragmented blocks, would further this goal. Future studies might benefit from using PacBio or other long read sequencing methodologies better able to assemble the repetitive sequence of the B chromosome.

## 5. Conclusions

Using a coverage ratio analysis, we were able to identify the sequence of a significant portion of the B chromosome in several cichlid species from Lake Malawi. An evaluation of this approach, including the comparison of sequence data types, has provided crucial insight for the future application of coverage ratio analysis to study B chromosomes in other taxa. The mapping of B blocks to their A chromosome homologs provides further support for the theory that B chromosomes collect sequences from the A genome. Both the rate of turnover and pattern of B blocks across the A genome provide important caveats to efforts to characterize the origin of B chromosomes. Finally, we identify a list of candidate genes and gene fragments located on the B chromosome that may include the gene(s) responsible for drive and female sex bias in Lake Malawi cichlids.

## Figures and Tables

**Table 1:** Sample Information.

Genus	Species	Locality	Sex	B?	Sample Type (#)	Sample ID	Sequencing Method	Mean Sequencing Depth
*Labeotropheus*	*trewavasae*	Thumbi	Female	B	Individual	2005-1306	Illumina	15.02
			Male	NoB	Pooled (10)	2005	Illumina	12.66
		Maison	Male	NoB	Pooled (20)	2012	Illumina	36.64
			XX Female	NoB	Pooled (20)	2012	Illumina	38.62
			WZ Female	NoB	Pooled (20)	2012	Illumina	36.63
*Melanochromis*	*auratus*		Female	B	Individual	2008-1601	Illumina	14.54
			Male	NoB	Pooled (10)	2005	Illumina	13.18
*Metriaclima*	*greshakei*		Female	B	Individual	2012-3493	Illumina	14.59
			Male	NoB	Pooled (20)	2012	Illumina	24.51
	*lombardoi*		Female	B	Individual	2014-1018	Illumina	16.21
			Female	B	Individual	2014-1021	Illumina	17.12
			Female	B	Individual	2014-1108	Illumina	11.75
			Female	B	Individual	2016-1012	PacBio	17.08
	*mbenji*		Female	B	Individual	2012-3997	Illumina	14.57
			Male	NoB	Pooled (20)	2012	Illumina	29.70
	*zebra*	Boadzulu	Female	B	Individual	2005-0976	Illumina	15.24
			Female	B	Individual	2005-0983	Illumina	14.78
			Female	B	Individual	2005-0986	Illumina	12.48
			Male	NoB	Pooled (20)	2012	Illumina	24.57
		Mazinzi	Male	NoB	Individual	SAMN03890374	PacBio	52.42
		Nkhata Bay	Female	B	Individual	2012-5340	Illumina	13.27
			Female	B	Individual	2012-5347	Illumina	16.20
			Male	NoB	Pooled (20)	2012	Illumina	34.39

**Table 2:** B Block Sizes.

	% of A Genome Passing Both Thresholds	Number of Blocks	Mean Block Size (bp)	Standard Deviation of Block Size (bp)	Median Block Size (bp)	Maximum Block Size (bp)
*L. trewavasae* 2005-1306	0.59	3517	1554.8	2592.9	849	42941
*M. auratus* 2008-1601	0.34	2476	1415.7	1859.6	836	30172
*M. greshakei* 2012-3493	0.69	4392	1395.1	2618.8	805	52821
*M. lombardoi* 2014-1018	1.31	10918	1285.1	1845.8	824	63229
*M. lombardoi* 2014-1021	1.04	8251	1298.0	1954.9	822	63250
*M. lombardoi* 2014-1108	1.10	8684	1274.9	1902.3	809	63229
*M. mbenji* 2012-3997	0.68	4147	1344.7	2519.2	783	63230
*M. zebra* “Boadzulu” 2005-0976	0.85	5907	1264.9	2002.4	793	42941
*M. zebra* “Boadzulu” 2005-0983	0.79	5369	1238.5	2402.4	769	100567
*M. zebra* “Boadzulu” 2005-0986	0.84	5986	1228.8	2293.8	771	100079
*M. zebra* “Nkhata Bay” 2012-5340	0.64	4869	1419.0	2856.6	842	99928
*M. zebra* “Nkhata Bay” 2012-5347	0.89	7162	1420.9	2821.8	867	100026
*M. lombardoi* 2016-1012 (PacBio)	0.59	1904	2971.1	4569.8	1723	98620
*L. trewavasae* “Maison” XX females (control)	0.44	2125	714.0	243.4	642	3607
*L. trewavasae* “Maison” WZ females (control)	0.06	343	819.5	478.5	686	5198
Core blocks	N/A	622	2194.6	3582.8	937	32721

**Table 3:** Total estimated length of B sequence identified in the coverage ratio analysis. The total length of sequence identified in the reference genome, or “in A space”, is provided for all B individuals, the two NoB controls, and the core block set (the blocks shared by at least 12 of the 13 B individuals). The total length of these blocks, after accounting for their copy number on the B chromosome (i.e., “in B space”) is provided for the 13 B individuals and 2 NoB controls. Similarly, the total length of just the core blocks, accounting for copy number, are provided for the 13 B individuals and 2 NoB controls. Finally, the portion of the total estimated B chromosome length that is represented by the core blocks is provided for the 13 B individuals.

	In A Space (Mb)	In B Space (Mb)	Core Blocks in B Space (Mb)	Core Blocks % of Total B in B Space
*L. trewavasae* 2005-1306	5.48	49.44	25.13	50.84
*M. auratus* 2008-1601	3.51	23.19	12.31	53.11
*M. greshakei* 2012-3493	6.14	58.20	30.84	52.98
*M. lombardoi* 2014-1018	14.06	74.53	24.09	32.31
*M. lombardoi* 2014-1021	10.73	62.58	23.00	36.76
*M. lombardoi* 2014-1108	11.09	58.48	17.67	30.22
*M. mbenji* 2012-3997	5.59	41.30	21.62	52.34
*M. zebra* “Boadzulu” 2005-0976	7.49	59.48	30.86	51.88
*M. zebra* “Boadzulu” 2005-0983	6.66	51.06	27.38	53.63
*M. zebra* “Boadzulu” 2005-0986	7.37	49.55	23.44	47.31
*M. zebra* “Nkhata Bay” 2012-5340	6.92	48.61	21.58	44.40
*M. zebra* “Nkhata Bay” 2012-5347	10.19	99.69	44.07	44.21
*M. lombardoi* 2016-1012 (PacBio)	5.66	35.40	15.02	42.44
*L. trewavasae* “Maison” XX females (control)	1.52	2.15	0.63	-
*L. trewavasae* “Maison” WZ females (control)	0.28	0.39	0.80	-
Core blocks	1.37	-	-	-

**Table 4:** Genes and gene fragments on the B chromosome.

Sample	Number of Genes and Gene Fragments
*L. trewavasae* 2005-1306	702
*M. auratus* 2008-1601	516
*M. greshakei* 2012-3493	972
*M. lombardoi* 2014-1018	2030
*M. lombardoi* 2014-1021	1688
*M. lombardoi* 2014-1108	1664
*M. mbenji* 2012-3997	899
*M. zebra* “Boadzulu” 2005-0976	1291
*M. zebra* “Boadzulu” 2005-0983	1262
*M. zebra* “Boadzulu” 2005-0986	1260
*M. zebra* “Nkhata Bay” 2012-5340	1094
*M. zebra* “Nkhata Bay” 2012-5347	1739
*M. lombardoi* 2016-1012 (PacBio)	678
*L. trewavasae* “Maison” XX females (control)	595
*L. trewavasae* “Maison” WZ females (control)	132
Core blocks	132

## Supplementary Materials

The following are available online at http://www.mdpi.com/2073-4425/9/12/610/s1.
